# A Three-Dimensional Hydrophobic Surface-Enhanced Raman Scattering Sensor via a Silver-Coated Polytetrafluoroethylene Membrane for the Direct Trace Detection of Molecules in Water

**DOI:** 10.3390/bios14020088

**Published:** 2024-02-05

**Authors:** Guanwei Tao, Jiajun Li, Yunyun Mu, Xinping Zhang

**Affiliations:** Institute of Information Photonics Technology, Beijing University of Technology, Beijing 100124, China

**Keywords:** SERS sensors, three-dimensional, hydrophobic, ultra-sensitive detection, aqueous solutions, PTFE membranes

## Abstract

We report a three-dimensional (3D) SERS substrate consisting of a silver nanoparticle (AgNP) coating on the skeleton-fiber surfaces of a polytetrafluoroethylene (PTFE) membrane. Simple thermal evaporation was employed to deposit Ag onto the PTFE membrane to produce grape-shaped AgNPs. The 3D-distributed AgNPs exhibit not only strong localized surface plasmon resonance (LSPR) but also strong hydrophobic performance. High-density hotspots via silver nano-grape structures and nanogaps, the large 3D interaction volume, and the large total surface area, in combination with the hydrophobic enrichment of the specimen, facilitate high-sensitivity sensing performance of such a SERS substrate for the direct detection of low-concentration molecules in water. An enhancement factor of up to 1.97 × 10^10^ was achieved in the direct detection of R6G molecules in water with a concentration of 10^−13^ mol/L. The lowest detection limit of 100 ppt was reached in the detection of melamine in water. Such a SERS sensor may have potential applications in food-safety control, environmental water pollution monitoring, and biomedical analysis.

## 1. Introduction

Surface-enhanced Raman scattering (SERS) spectroscopy [1,2] can quickly and precisely identify target molecules via the “fingerprint” Raman spectrum with high sensitivity. This makes SERS spectroscopy a powerful detection technique in the fields of food safety, environmental science, pharmaceutical, and biological testing [3,4,5,6]. Localized surface plasmon resonance (LSPR) is the basic physics for SERS, where hotspots as strongly enhanced photoelectric fields locally generated on metallic nanostructures [7,8,9,10]. LSPR performance can be precisely controlled by designing metallic nanostructures of different sizes [11,12], different shapes [13,14], and different materials [15,16]. Although there are extensive reports on the design and realization of SERS substrates in different forms, there are always increasing demands for sensors with more versatile utility and more general applications. In particular, sensors for the direct detection of low-concentration molecules in the gas or liquid phase are urgently needed.

Most SERS sensors employ planar hydrophilic substrates so that the interaction area between the local field and the specimen molecules is limited to the focusing laser spot. Furthermore, the hydrophilic substrate is usually affected by the coffee ring effect. The target molecules in the solution are easily diffused into the non-excited region, resulting in a decreased number of molecules for detection and reducing both the sensitivity and the homogeneity of the device. The construction of a three-dimensional (3D) hydrophobic SERS substrate is more meaningful in the condensation of target molecules [17,18,19,20,21]. Moreover, the 3D structure can expand the interaction volume and total surface area between the local field and the target molecules [22], improving the SERS performance substantially. For the preparation of SERS substrate, the chemical synthesis method has been widely used because of its high speed and low costs [23]. However, chemical synthesis usually requires surfactants or reducing agents that will blunt SERS activity [24]. In addition, many chemical-reducing agents are toxic and not environmentally friendly. In contrast, uniform nanostructures can be prepared over large areas using physical vapor deposition (PVD), which is environmentally friendly. For example, M. Gilic et al. prepared a 3D SERS sensor to detect R6G by gilding a film on diatoms [25]. Polytetrafluoroethylene (PTFE) has stable physical and chemical properties, excellent thermal stability, acid and alkali resistance, and is an excellent candidate for SERS substrates.

Exploring SERS sensors for the detection of melamine (1,3,5-triazine-2,4,6-triamine) is of great importance for food safety since it was illegally added to liquid foods ingested by infants, which may lead to serious health problems and even threaten human lives [26]. However, the currently available detection techniques, including gas- [27] or liquid- [28] chromatography–mass spectrometry, inductively coupled plasma–mass spectrometry [29], and enzyme-linked immunoassay (ELISA) [30], have limitations such as high analysis expenses, time-consuming procedures, complex sample preparation, and large consumption of reagents. Therefore, the simple, rapid, and stable SERS sensing technique with high specificity and high sensitivity is the most suitable candidate to replace the conventional techniques in the detection of melamine.

In this work, we demonstrate an economical and simple method for accomplishing a high-sensitivity SERS substrate, where a PTFE membrane is employed as the platform for accomplishing the plasmonic nanostructures. The PTFE membrane has high physical/chemical stability and high-density 3D microstructures that are very suitable for constructing SERS substrates. Furthermore, using PTFE membrane as the platform, we were able to produce 3D-distributed silver nanoparticles (AgNPs) in one step via thermal evaporation without any subsequent annealing treatment. The AgNP exhibit grape shapes and are densely distributed in the third dimension (3D), implying a high density of the SERS hotspots. Moreover, such a SERS substrate exhibits strong hydrophobic properties, which facilitates strong enrichment of the target molecules on the hotspots. High simplicity in the structural design and the fabrication procedures, low costs, and high flexibility with easily tailorable shapes and sizes are the apparent advantages of this present SERS substrate. Large 3D interaction volume and large total surface area constitute further advantages of such a sensing device. In the detection of melamine in its aqueous solution, we achieved an ultra-high sensitivity of 100 ppt (0.1 ppb or 10^−4^ ppm).

## 2. Construction and Characterization of the Ag-PTFE SERS Sensor

**Figure 1:**
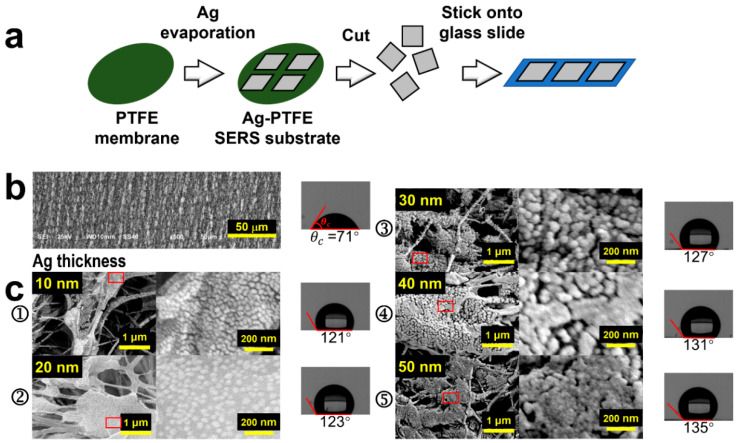
(**a**) Schematic illustration of the fabrication process of the Ag-PTFE SERS substrates. (**b**) SEM image of the PTFE before being coated with Ag with measurement on the contact angle of water. (**c**) SEM images of the Ag-coated PTFE membrane with different thicknesses of Ag and measurements on the contact angles of water (➀ 10, ➁ 20, ➂ 30, ➃ 40, and ➄ 50 nm).

Figure 1a demonstrates the preparation of Ag-PTFE SERS substrates. The PTFE membrane was first rinsed with anhydrous ethanol and DI water before it was dried. Silver was then thermally evaporated onto the PTFE membrane, where a mask defining square evaporation area was employed. The vacuum thermal evaporator is equipped with a film thickness monitoring and display system. We determined the thickness of the Ag layer over the PTFE membrane by the number displayed on the film thickness monitor, which is a relatively reliable value. The Ag deposition has a varied thickness in order to optimize the SERS performance of the device. The prepared Ag-PTFE SERS substrate was flexible and could be cut into suitable sizes and pasted on a glass slide, as shown on the right hand of Figure 1a.

Figure 1b shows the SEM image of the PTFE membrane, where 3D textures in micro-scales can be clearly observed, which is exactly the basis for constructing the 3D SERS substrate. In Figure 1c, we show the SEM images of Ag-coated PTFE substrates with the Ag thickness varied from 10 to 50 nm, as shown by Figure 1c(➀–➄), respectively. Meanwhile, we included the contact-angle measurements of a water drop on these substrates on the right hand of Figure 1b,c, which demonstrates a significant increase in the hydrophobicity of the substrate with the deposition of silver. Silver nanoparticles with varied sizes and varied gap widths are produced on the surface of the PTFE membrane. The gaps between the silver nanoparticles with different gap widths and different depths, as well as the morphological variation with PTFE surface structures, are the main mechanisms responsible for the increased hydrophobicity, which is supported by the Cassie–Baxter model [31]. Apparently, the deposition of Ag produces closely arranged nanoparticles, instead of continuous films, on the surface of the 3D textures and the size of the Ag nanoparticles (AgNPs) is increased with increasing the thickness of the deposition. However, when the thickness reaches 50 nm, the AgNPs tend to get connected to form pieces of continuous film in different locations. Due to the variation of the surface morphology of the PTFE textures, the density of the AgNPs also varies with locations, which defines volume SERS with a global enhancement in practical applications. Figure S1a,b provide schematic illustrations for the atomic-scale growth dynamics during the thermal evaporation of Ag onto a high- and low-surface-energy substrate, respectively. For a high-energy surface, evaporated silver atoms hit the surface and diffuse randomly. Due to the limited kinetic energy, incoming atoms form new clusters or diffuse to edge locations of nearby existing clusters. The clusters will merge to form island structures, implying a much higher lateral growth rate than the vertical. Although gaps may still exist between discrete islands, the gap density will be very limited. However, for a low-energy surface, the thermodynamic driving force due to the surface energy difference between the low-energy surface and the metal atoms drives the atoms to diffuse along the metal cluster surface. The vertical growth rate of clusters increases while the lateral decreases. Finally, high-density “nanograpes” with narrow gaps will be produced. The growth and morphology of silver nanostructures were controlled kinetically and thermodynamically by adjusting the deposition parameters.

More specifically, in the early stage of deposition, many small silver nanoparticles with gaps appear on the surface of the PTFE fiber, but the vertical height is small. When the deposition thickness gradually increases from 10 to 30 nm, the silver nanoparticles continue to grow, forming a rich and dense grape-like silver nanostructure with narrow gaps instead of an island structure. The resulting dense and narrow gaps can act as hot spots. However, when the thickness reaches 40 nm, some of the adjacent silver nanograpes merge to form an island-like structure, and it is increased further to 50 nm, most of the adjacent silver nanograpes merge into silver nanoislands due to the expansion of the clusters, so that the gaps almost disappear. We used the same deposition rate of 0.2 Å/s for all of the fabricated structures. The deposition thickness of 10, 20, 30, 40, and 50 nm corresponds to the deposition time of 500, 1000, 1500, 2000, and 2500 s, respectively. Figure S2a–c provide the corresponding size distribution analysis when the deposition thickness is 10, 20, and 30 nm, respectively. Because many particles expand to form island structures when the deposition thickness is 40 and 50 nm, the corresponding size distribution analysis was not carried out.

We attribute this growth mechanism to the large difference in surface energy between the substrate and the silver atomic vapor with a surface energy of γ_Ag_ = 1500 mJ/m^2^ [32], corresponding to the island growth mechanism by the Volmer–Weber model [33]. Such thermal growth mechanisms have also been reported for gold nanopearls on substrates with ultralow surface energy [18]. To verify the low-surface-energy properties of PTFE, we measured the contact angles of both water and n-hexadecane on the pure PTFE membrane, as shown in Figure S3, which are 71° and 20°, respectively. Thus, the surface energy of the PTFE membrane can be calculated by Owens’ method [34] as follows:

(1) $$\left(cos\theta +1\right)\gamma_{L}=2\sqrt{\gamma_{S}^{d}\gamma_{L}^{d}}+2\sqrt{\gamma_{S}^{p}\gamma_{L}^{p}},$$

where θ is the contact angle; $\gamma_{S}^{d}$ and $\gamma_{S}^{p}$ are the dispersion force and the polar force for the solid, respectively; $\gamma_{L}^{d}$ and $\gamma_{L}^{p}$ are the dispersion force and polar forces for the liquid, respectively; and $\gamma_{L}$ is the surface tension of the liquid. The values of $\gamma_{L}^{d}$ and $\gamma_{L}^{p}$ for water and n-hexadecane are given in Table S1. Therefore, the calculations using (1) produce $\gamma_{PTFE}^{d}=25.9\;mJ\;m^{-2}$ and $\gamma_{PTFE}^{p}=11.9\;mJ\;m^{-2}$ for the PTFE membrane. Consequently, the surface energy of the PTFE membrane can be obtained using formula $\gamma_{PTFE}=\gamma_{PTFE}^{d}+\gamma_{PTFE}^{p}=37.8\;mJ\;m^{-2}$, which is nearly 40 times lower than that of silver, verifying the large surface energy difference between PTFE and atomic vapor of Ag.

The most prominent feature of the AgNP-coated PTFE membrane is its hydrophobic transition. The PTFE membrane supplies a hydrophilic surface, as shown in the right hand of Figure 1b, although a large contact angle of 71° was measured for water. However, after being coated with Ag, all of the substrates become hydrophobic, as shown in the right-hand pictures of Figure 1c, where a contact angle of 121°, 123°, 127°, 131°, and 135° was measured for the Ag-coating thickness of 10, 20, 30, 40, and 50 nm, respectively. Therefore, the AgNP structuring modified the surface performance of the PTFE membrane completely. The hydrophobic properties facilitate strong condensation of the molecules in solution specimens, enabling enhanced SERS signals and detection sensitivity.

## 3. Optimization of the SERS Performance by the Ag-Coated PTFE Membrane

Figure S4 shows a Raman detection system. The output of a fiber-coupled 785 nm laser is re-collimated (by lens L1) before passing through a 785 nm band-pass filter, ensuring a narrow-band excitation laser source at 785 nm. It is then reflected by a dichroic mirror and focused onto a SERS substrate (by lens L2). The Raman scattering signal is re-collimated by L2 and passes through the dichroic mirror and a long-pass filter to filter out the excitation laser. The signal is ultimately focused (by lens L3) into the detector head of the fiber-coupled Raman spectrometer. In all of the measurements, 1 μL sample solution was added to the Ag-SERS substrate dropwise using a pipette.

**Figure 2:**
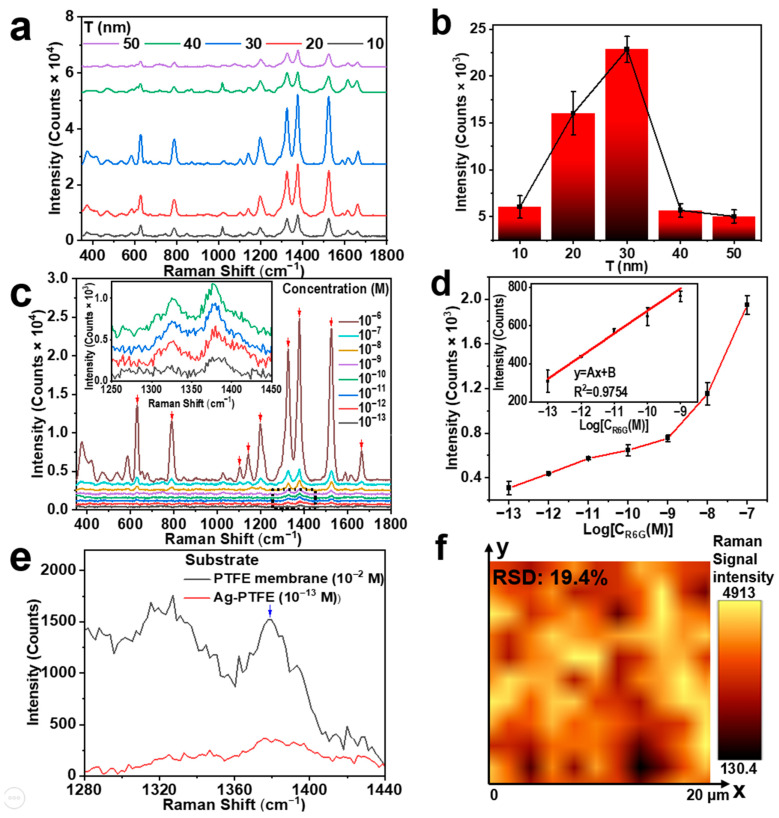
(**a**) SERS measurements on R6G/water solution with a concentration of 10^−6^ M for the Ag-PTFE substrates with different Ag thickness. (**b**) Diagram of the SERS signal intensity at 1378 cm^−1^ measured on R6G/water solution with a concentration of 10^−6^ M for different thicknesses of Ag coating. (**c**) SERS spectra measured on R6G/water solution with different concentrations (10^−13^ M–10^−6^ M) using Ag-PTFE SERS substrate with an Ag thickness of 30 nm. (**d**) A plot of the SERS signal intensity at 1378 cm^−1^ as a function of solution concentration. Inset: linear relationship between the SERS signal intensity and R6G/water concentration in the range of 10^−13^–10^−9^ M. (**e**) A comparison between the Raman spectra measured using a pure PTFE membrane and using an Ag-coated PTFE on the R6G/water solution with a concentration of 10^−2^ M (black) and 10^−13^ M (red), respectively. (**f**) A 3D mapping within an area of 20 × 20 μm^2^ of the SESRS signal intensity measured on the R6G/water solution with a concentration of 10^−6^ M.

As illustrated in Figure 1c, the evaporation thickness of Ag is very crucial for the surface morphology, the hydrophobic properties, and, consequently, the SERS performance of the Ag-PTFE substrate. Using a typically low concentration of the R6G/water solution as the detection target, we measured the SERS signals by Ag-PTFE substrates with different Ag evaporation thicknesses, as shown in Figure 2a.

In the measurements on the R6G/water solution with a concentration of 10^−6^ M, a laser intensity of 184 W/cm^2^ at 785 nm and an integration time of 15 s was employed. Data acquisition was carried out within 3–10 min after the sample liquid was dropped onto the substrate. The obtained SERS spectra were processed by the asymmetric least squares smoothing (ALSS) algorithm. As shown in Figure 2a, the main Raman peaks of R6G are observed at 631, 787, 1102, 1142, 1197, 1325, 1378, 1525, and 1664 cm^−1^. The above conditions also apply to all measurements in the following sections. We can find that the intensity of the signal spectrum increases at all Raman peaks with the Ag evaporation thickness (T = 10~50 nm) within the first 30 nm and starts to decrease for Ag thickness larger than 30 nm. As summarized in Figure 2b, the Raman signal at 1378 cm^−1^ reaches its peak intensity at a Ag thickness of 30 nm. This corresponds to the grape-like silver nanostructure with a dense narrow gap in Figure 1c(➂), where the nanostructure has the highest hot-spot density, and the enhancement results are consistent with the structures. This justifies the optimized thickness of Ag coating, and the 30 nm thickness is employed for all of the following SERS measurements.

Figure 2c shows the SERS measurement results on R6G aqueous solutions with concentration reduced from 10^−6^ to 10^−13^ M. The main Raman peaks are indicated by downward red arrows. The signal at 1378 cm^−1^ has the highest peak intensity and can still be observed at 10^−13^ M with excellent contrast. Figure 2d plots the relationship between the peak intensity of the SERS signal at 1378 cm^−1^ as a function of the logarithmic value of the R6G/water concentration. A linear relationship is observed for concentrations ranging from 10^−13^ to 10^−9^ M, implying a linear correlation of the peak intensity at 1378 cm^−1^ with the logarithmic value of the concentration with a correlation coefficient of R^2^ = 0.9816. This justifies the sensitivity performance with the excellent response of the Ag-PTFE SERS substrate for the direct detection of low-concentration molecules in water. However, a different slope can be measured for the data at concentrations higher than 10^−9^ M, indicating different SERS behaviors of the substrate for low and high concentrations of the sample. In particular, when the concentration reaches 10^−6^ M, the signal intensity is too high to be included suitably in the display of Figure 2d and is thus not included in the plot.

We use the following formula to calculate the SERS enhancement factor (EF) of the Ag-PTFE SERS substrate:

(2) $$EF=\frac{I_{SERS}\times C_{RS}}{I_{RS}\times C_{SERS}},$$

where I_RS_ and I_SERS_ are the intensities of Raman signals measured using the PTFE membrane without and with Ag coating, respectively, and C_RS_ and C_SERS_ are the corresponding solution concentrations, respectively. The enhancement factors of SERS substrates with deposition thicknesses of 10, 20, 30, 40, and 50 nm are given in Table S2. Figure 2e shows the Raman spectrum of R6G aqueous solution with a concentration of 10^−2^ M added directly on the PTFE membrane (black) and the SERS spectrum of R6G aqueous solution with a concentration of 10^−13^ M added on the Ag-PTFE SERS substrate (red). The EF value can be calculated to be about 1.97 × 10^10^, verifying the ultra-sensitivity performance of the Ag-PTFE SERS sensor.

The homogeneity of the Ag-PTFE SERS substrate can be verified by the 3D mapping of the Raman signals measured on the aqueous R6G solution with a concentration of 10^−6^ M, as shown in Figure 2f. Clearly, over the whole area of 20 × 20 μm^2^, the intensity of the Raman signals ranges from about 130 to 4900 counts, which is a reasonably excellent homogeneity for such a 3D SERS substrate. The optical image of the mapped area is given in Figure S5.

To evaluate the reliability and stability of the Raman signals measured using the Ag-PTFE substrate, we carried out test experiments for the detection of R6G aqueous solution with a concentration of 10^−3^ M over a time range of 0.5–30 min, where other conditions were kept unchanged. Figure S6 plots the relative Raman signal intensity as a function of time, where the relative intensity is defined as by I(t)/I_0_ in percentage. I(t) is the Raman signal intensity at 1378 cm^−1^ as a function of time (t), and I_0_ is the maximum value of Raman signal intensity at 1378 cm^−1^ within the first 30 min. The sample solution needs time to penetrate completely into the 3D structures of the PTFE membrane. As shown in Figure S5, this time duration is roughly 3 min. The detected Raman signal reaches its maximum shortly after the first 3 min. However, the Raman signal obviously decays after 10 min. Therefore, all our measurement results have been taken within the first 3–10 min after the dropping of the sample solution.

To avoid oxidization of Ag, the SERS substrate was stored in a glovebox with a nitrogen environment. The stability of the substrate was evaluated by observing the SERS signal change over 14 days, which was measured on R6G/water solutions with a concentration of 10^−6^ M. Each signal is an averaged value for five collections on five randomly selected locations on the substrate, as shown in Figure S7a. For Raman detection at 1378 cm^−1^, the signal is almost the same as the initial intensity, even after 14 days. As shown in Figure S7b, we measured the intensity of the Raman signal at 1378 cm^−1^ for five different locations on the substrate on days 1, 7, and 14. These three groups of data demonstrate an RSD value of only 4.8%. Thus, the substrate stored in a nitrogen environment can be used for a long time.

We summarize the SERS enhancement mechanisms as follows: (1) the 3D structures of the fiber textures in the PTFE membrane act as a supporting skeleton for the growth of AgNPs into nanograpes with narrow gaps during the thermal evaporation process; (2) the rough surface of the Ag nanograpes attaching to the PTFE textures produces strong localized surface plasmons, so that such a scheme supplies SERS hotspots with high density and high specific surface area; and (3) the hydrophobic performance of the Ag-PTFE SERS substrate enables condensation of the sample molecules during solution evaporation, which prevents the target molecules from diffusing into the non-detected region with the solution, ensuring effective enrichment within the detection region.

## 4. Detection of Melamine in Water

This SERS sensor exhibits excellent suitability for the detection of pollutant molecules in water or in liquid food. As a most practical application, melamine with extremely low concentration in water is detected using such an Ag-PTFE SERS substrate, where we reduced the concentration of the aqueous solution of melamine from 10^2^ ppm to 10^−4^ ppm. In the measurements, a drop of 1 μL melamine aqueous solution with a different concentration was added to the surface of the Ag-PTFE substrate for each measurement, and three measurements were carried out for each concentration for stability of the measurement results.

**Figure 3:**
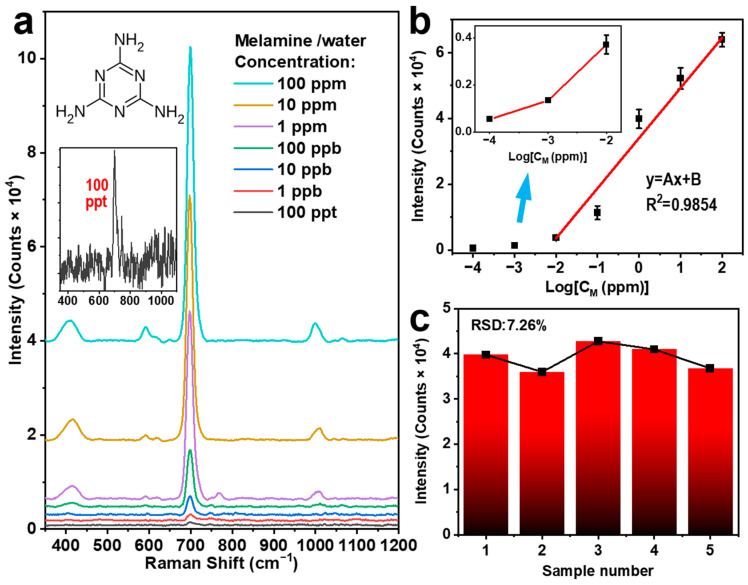
(**a**) SERS spectra measured on melamine aqueous solution with different concentrations (10^−4^ ppm–10^2^ ppm) using an Ag-PTFE SERS substrate with a Ag thickness of 30 nm. (**b**) The relationship between the SERS signal intensity at 700 cm^−1^ and melamine/water concentration. (**c**) The diagram of the SERS signal intensity at 700 cm^−1^ measured on the melamine aqueous solution (1 ppm) for 5 different batches of the Ag-PTFE SERS substrates shows stability of both the measurement results and the method.

A similar power of 25 mW (power density of about 180 W/cm^2^) as above and an integration time of 30 s were employed for the excitation laser 785 nm. Figure 3a shows the SERS spectra measured on the aqueous solution of melamine with different concentrations (10^−4^ ppm–10^2^ ppm) with the molecular structure of melamine included in the inset (upper panel). Characteristic Raman peak of melamine is observed at 700 cm^−1^, which exhibits the most intensive signal in the Raman spectrum. This signal can be attributed to the ring breathing vibrational mode of melamine, which is clearly observable with high contrast for a concentration of 10^−4^ ppm (or 100 ppt), as shown in the inset (lower panel). Figure 3b plots the intensity of the detected Raman signal as a function of the solution concentration. A linear relationship can be observed for concentrations ranging from 10^−2^ ppm to 10^2^ ppm. A different slope of the variation is observed for concentrations from 10^−4^ to 10^−2^ ppm, indicating the performance approaching the detection limit. We can confirm a detection limit lower than 10^−4^ ppm (100 ppt) for melamine-polluted water using our scheme of the Ag-coated PTFE, which justifies the high sensitivity of such a SERS sensor.

To evaluate the reproducibility of Ag-PTFE SERS substrates, we randomly selected five different batches of Ag-PTFE SERS substrates and carried out detection measurements on an aqueous solution of melamine with a concentration of 1 ppm. Figure 3c shows the peak intensity at 700 cm^−1^ of the measured Raman spectra as a function of the batch number. We can clearly observe a roughly constant SERS signal intensity at 700 cm^−1^ for different substrates, where an RSD value of only 7.26% was calculated, confirming excellent reproducibility and stability of the Ag-PTFE SERS substrates.

We make a comparison with the results reported in previous publications, as shown in Table 1. Apparently, our Ag-PTFE SERS substrate demonstrated in this work exhibits the best sensitivity with the highest enhancement factor. It can be fabricated on a large scale with an environmentally friendly process. This technique has the advantages of no need for surfactant or reducing agents during the preparation and no need for subsequent annealing process after the thermal evaporation. Apparently, the simplicity of the fabrication technique and low costs of the fabrication equipment, the employed materials, and the finished devices are the apparent advantages of the Ag-PTFE SERS substrates.

## 5. Conclusions

We developed an ultra-sensitive 3D hydrophobic SERS substrate by thermal evaporation Ag on the PTFE membrane with an optimized thickness. Making use of the large surface-energy difference between PTFE and Ag atomic vapor, Ag nanograpes are directly produced on the skeleton fibers of the PTFE membrane, which can be optimized by controlling deposition parameters without any other treatment. Direct detection of low-concentration R6G and melamine in molecules in water was achieved with high sensitivity, where a SERS enhancement factor of 1.9 × 10^10^ was achieved. The localized surface plasmon resonance of the 3D-distributed AgNPs with large volume and large surface area and the strong hydrophobic effect of Ag-PTFE substrate have been the most important mechanisms. In the detection of melamine in water, we achieved the highest detection sensitivity of 0.1 ppb, and the signal showed excellently high contrast in the range of 0.1 ppb–100 ppm. This developed SERS sensor will find extensive applications in the fields of liquid food safety and environmental water pollution monitoring.

## 6. Experimental Methods

### 6.1. Materials and Regents

Melamine (99%) was purchased from Alfa Aesar Chemical Co., Ltd. (Shanghai, China), Rhodamine 6G (R6G) from Shanghai Aladdin Bio-Chem Technology Co. (Shanghai, China) and PTFE membrane filter Tianjin Jinteng Technology Co., Ltd. (Tianjin, China), Deionized water (DI water) was purified via Thermo scientific D50282 from Thermo Fisher Scientific (Waltham, MA, USA) (18.2 MΩ · cm) and was employed for all aqueous solutions.

### 6.2. Characterizations

Scanning electron microscopic (SEM) images were acquired using a Merlin Compact at a voltage of 5 kV. A continuous-wave laser at 785 nm with a linewidth of 0.72 nm and a maximum output power of 500 mW from Shanghai Oceanhood Optoelectronics Tech Co. Ltd. (Shanghai, China), was used as the Raman excitation light source. A Raman spectrometer from Ocean Optics (QEP01609) with an effective spectral range of 0~2098 cm^−1^ and a resolution of 0.16 nm was used for the collection of Raman signals. 

### 6.3. Detection of R6G Molecules in Water

A solution of R6G (10^−2^ M) was prepared by mixing 47.9 mg R6G powder in 10 mL DI water while stirring. This solution was subsequently diluted in DI water to reach a series of concentrations. Pure DI water was used as the control sample.

### 6.4. Detection of Melamine Molecules in Water

An aqueous solution of melamine with a concentration of 100 ppm was first prepared by mixing 10 mg pure melamine powders into 100 mL DI water while stirring. This solution was subsequently diluted in DI water to reach a series of melamine standard solutions: 10, 1, 0.1, 10^−2^, 10^−3^, and 10^−4^ ppm. Pure DI water was used as the control group.

## Figures and Tables

**Table 1:** Comparison with previously reported results.

SERS Substrate	Sensitivity	EF	Ref.
AuNP/ZnO/ZnFe_2_O_4_	0.39 μM	1.61 × 10^8^	[35]
Ag/rGO nanohybrid	10^−8^ M	8.4 × 10^6^	[36]
HARNPA/Ag	5.6 × 10^−7^ M	1.44 × 10^7^	[37]
Ag-PTFE	0.1 ppb	1.97 × 10^10^	This work

## Data Availability

Data are contained within the article and supplementary materials.

## Supplementary Materials

The following supporting information can be downloaded at https://www.mdpi.com/article/10.3390/bios14020088/s1. Figure S1: Atomic growth mechanisms for the evaporation of Ag onto surfaces with high (a) and low (b) surface energies; Figure S2: Size distribution analysis of AgNPs with deposition rate of 12 nm/min and deposition thickness of (a) 10 nm, (b) 20 nm, and (c) 30 nm; Figure S3: Contact Angle measurements of DI water (a) and n-Hexadecane (b) on pure PTFE membrane; Figure S4: Optical setup for the Raman detection using the Ag-coated PTFE membrane; Figure S5: The optical microscopic image of the mapped area; Figure S6: Raman intensity at 1378 cm^−1^ in R6G aqueous solution (10^−3^ M) was measured on Ag-PTFE SERS substrates within 0.5–30 min; Figure S7: (a) The variation of SERS intensity measured at 1378 cm^−1^ on the sample stored in nitrogen over 14 days. (b) The SERS signal intensity measured on 15 points at 1378 cm^−1^ on day 1 (empty), 7 (yellow), and 14 (blue) with 5 measurements on each day; Table S1: Surface tension of liquids; Table S2: EF of SERS substrates.
