# Supplementary material for: A Three-Dimensional Hydrophobic Surface-Enhanced Raman Scattering Sensor via a Silver-Coated Polytetrafluoroethylene Membrane for the Direct Trace Detection of Molecules in Water
Source: Biosensors (Basel). 2024 Feb 5;14(2):88. doi: 10.3390/bios14020088 (PMC10886991; doi:10.3390/bios14020088)
Supplement: Supplementary file 1 [file biosensors-14-00088-s001.zip › biosensors-2806696-supplementary.pdf]

Supporting information

A 3D hydrophobic SERS substrate by silver-coating PTFE membrane for sensitive detection of melamine

Guanwei Tao, Jiajun Li, Yunyun Mu, and Xinping Zhang*

Institute of Information Photonics Technology, Beijing University of Technology,

Beijing 100124, P. R. China

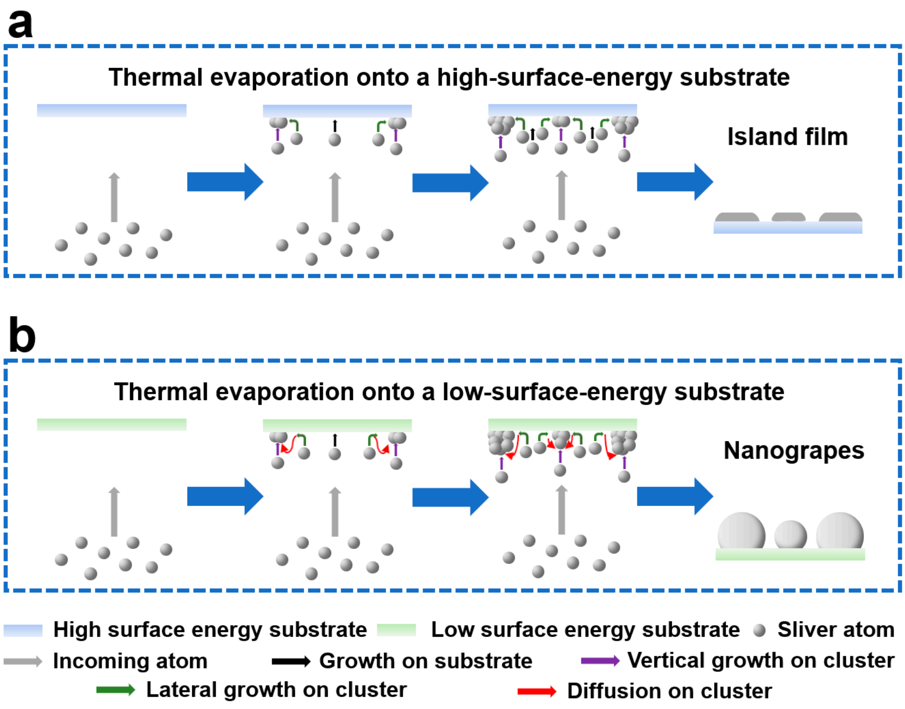

Figure S1 Atomic growth mechanisms for the evaporation of Ag onto surfaces with high (a) and low (b) surface energies.

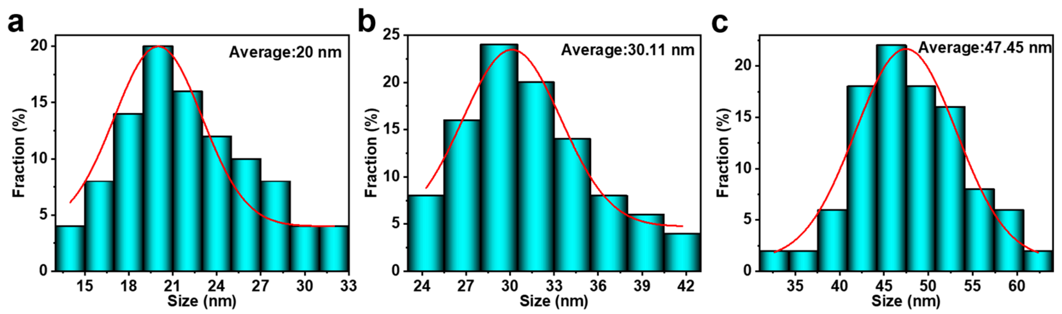

Figure S2 Size distribution analysis of AgNPs with deposition rate of 12 nm/min and deposition thickness of (a) 10 nm, (b) 20 nm, and (c) 30 nm.

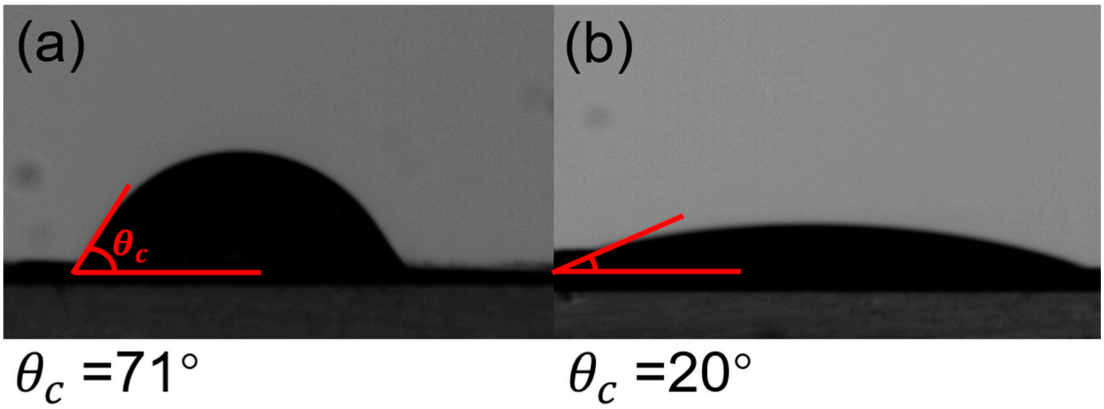

Figure S3 Contact Angle measurements of DI water (a) and n-Hexadecane (b) on pure PTFE membrane.

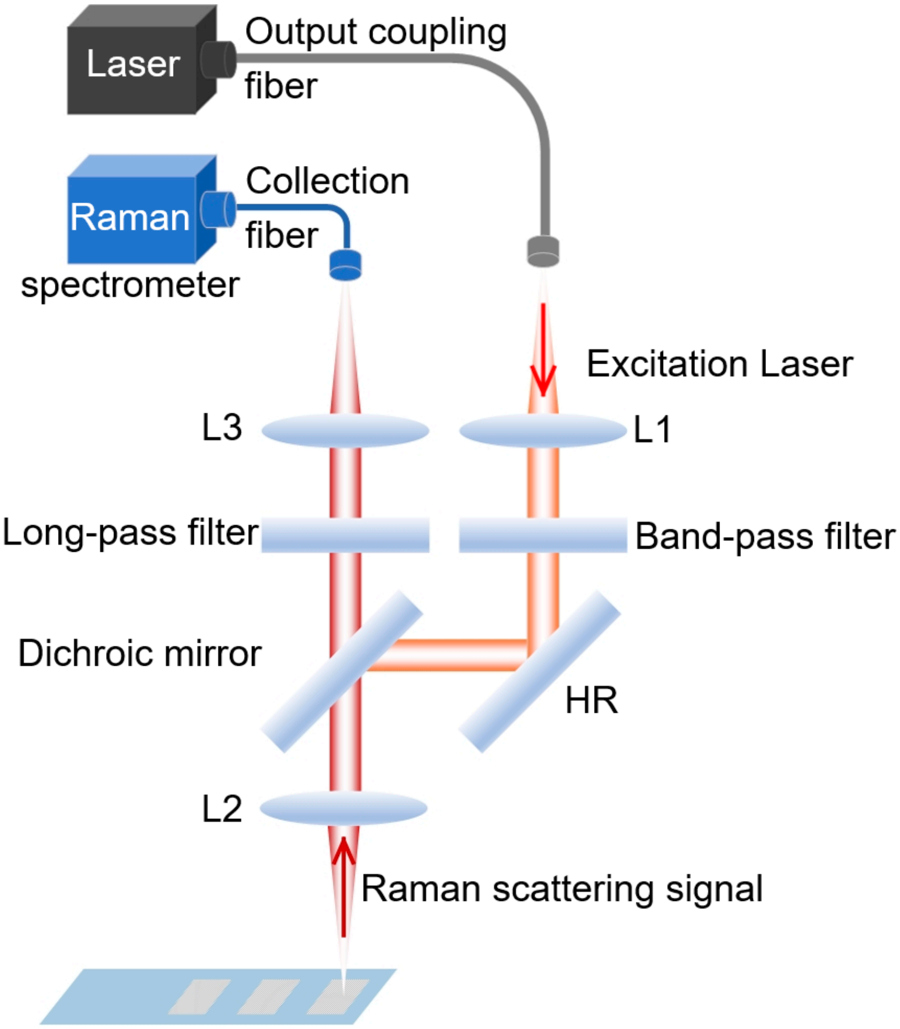

Figure S4 Optical setup for the Raman detection using the Ag-coated PTFE membrane.

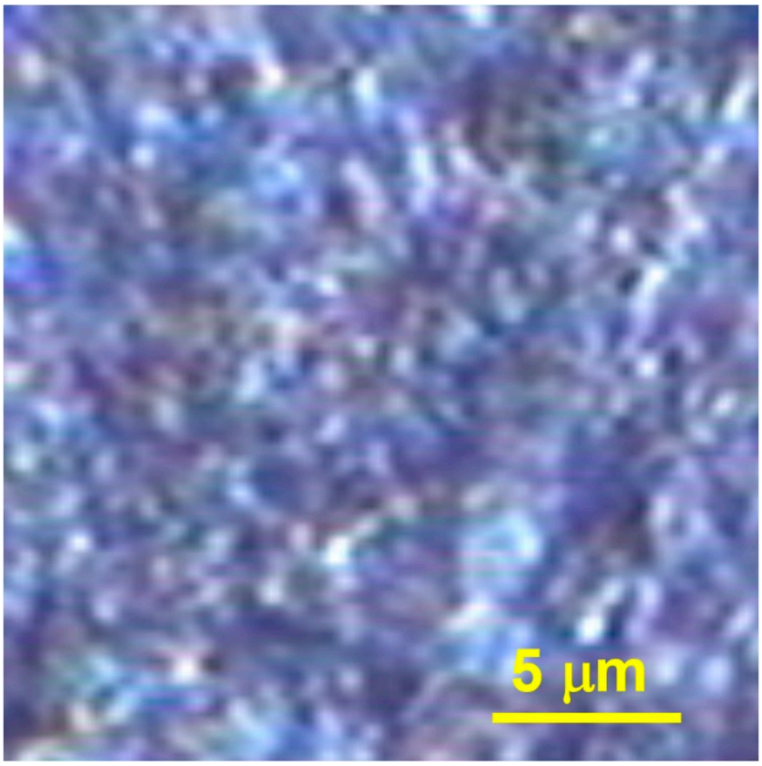

Figure S5 The optical microscopic image of the mapped area.

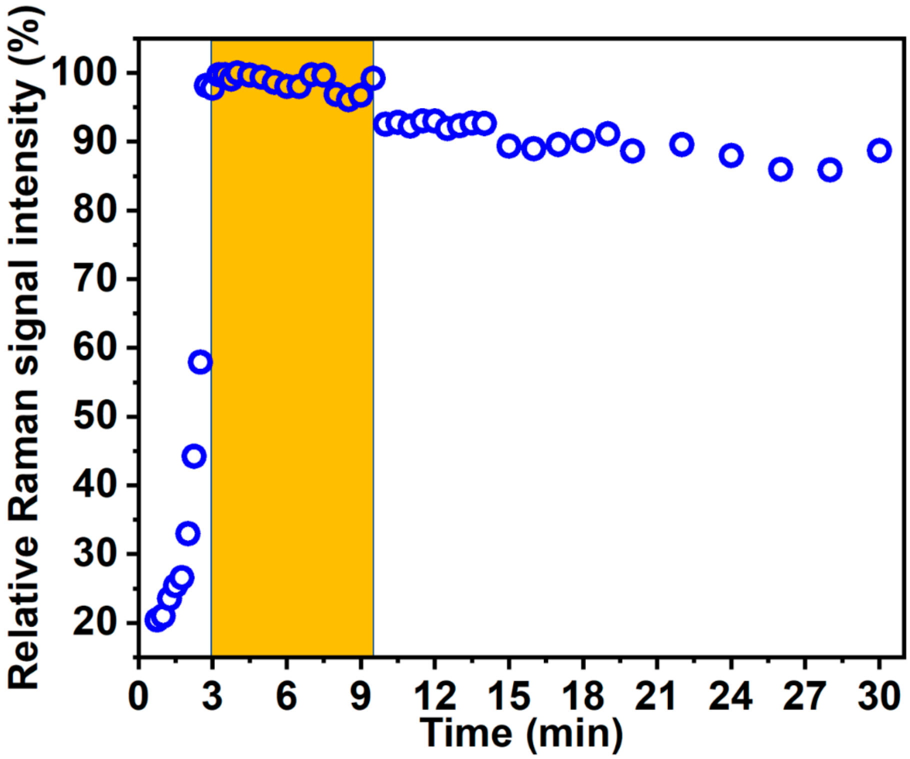

Figure S6 Raman intensity at 1378 cm^{-1} in R6G aqueous solution (10^{-3} M) was measured on Ag-PTFE SERS substrates within 0.5-30min.

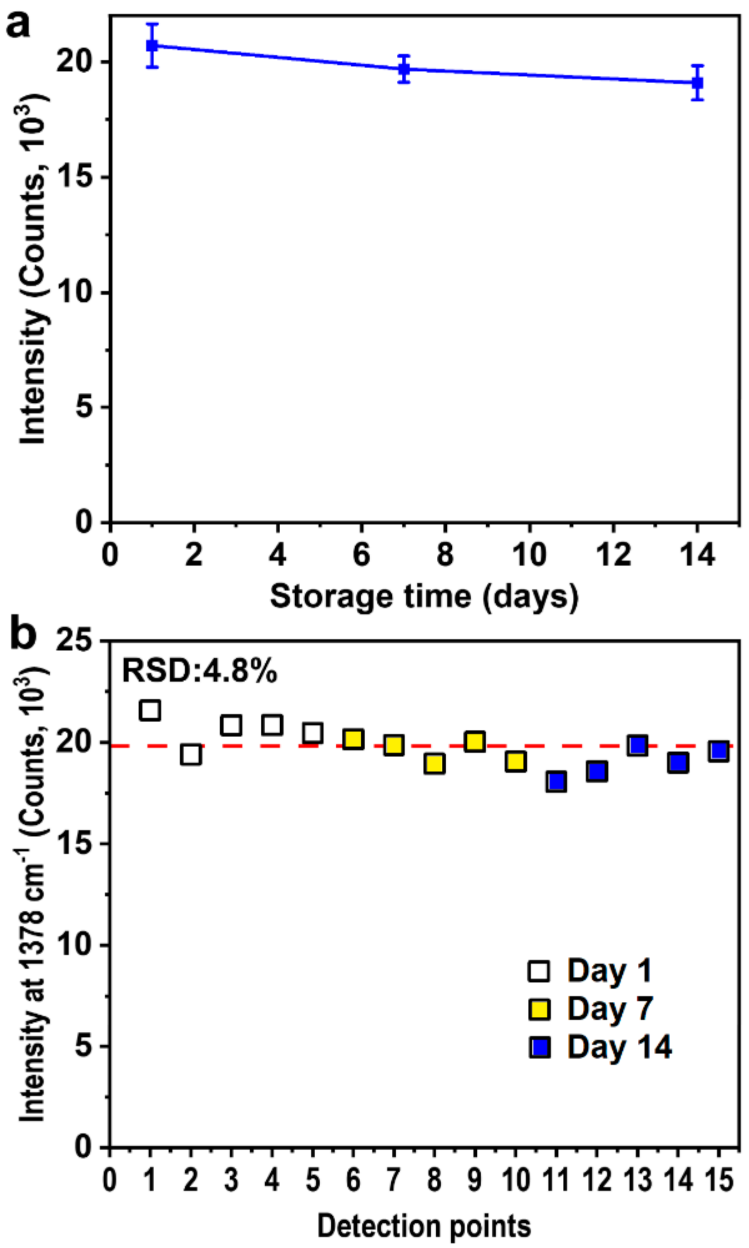

Figure S7 (a) The variation of SERS intensity measured at 1378 cm^{-1} on the sample stored in nitrogen over 14 days. (b) The SERS signal intensity measured on 15 points at 1378 cm^{-1} on day 1 (empty), 7 (yellow), and 14 (blue) with 5 measurements on each day.

Table S1

Surface tension of liquids

Liquid	γ_L^p (mN·m^{-1})	γ_L^d (mN·m^{-1})	γ_L (mN·m^{-1})	γ_L^p/γ_L^d	Characteristics
Water	51	21.8	72.8	2.36	Polar
n-Hexadecane	0	27.6	27.6	0	Non-polar

Table S2

EF of SERS substrates

Deposition thickness (nm)	C_{SERS} (M)	I_{SERS}	EF
10	10^{-6}	7589	6.69×10^{4}
20	10^{-6}	18489	1.65×10^{5}
30	10^{-13}	223	1.97×10^{10}
40	10^{-6}	7193	6.43×10^{4}
50	10^{-6}	5806	5.19×10^{4}
